# Monitoring and Validation of the Global Replacement of tOPV with bOPV, April–May 2016

**DOI:** 10.1093/infdis/jiw558

**Published:** 2017-06-30

**Authors:** Margaret Farrell, Lee M. Hampton, Stephanie Shendale, Lisa Menning, Alejandro Ramirez Gonzalez, Julie Garon, Samantha B. Dolan, Gaël Maufras du Châtellier, Sarah Wanyoike, Diana Chang Blanc, Manish M. Patel

**Affiliations:** 1 UNICEF Program Division, New York;; 2 Global Immunization Division, Centers for Disease Control and Prevention, Atlanta, Georgia;; 3 Expanded Programme on Immunization, World Health Organization, Geneva, Switzerland;; 4 Emory University, Atlanta, Georgia;; 5 West and Central Africa Regional Office, UNICEF,Dakar, Senegal; and; 6 Task Force for Global Health, Atlanta, Georgia

**Keywords:** OPV, polio, switch, eradication, monitoring.

## Abstract

The phased withdrawal of oral polio vaccine (OPV) associated with the Polio Eradication and Endgame Strategic Plan 2013-2018 began with the synchronized global replacement of trivalent OPV (tOPV) with bivalent OPV (bOPV) during April - May 2016, a transition referred to as the “switch.” The World Health Organization’s (WHO) Strategic Advisory Group of Experts (SAGE) on Immunization recommended conducting this synchronized switch in all 155 OPV-using countries and territories (which collectively administered several hundred million doses of tOPV each year via several hundred thousand facilities) to reduce risks of re-emergence of vaccine-derived polioviruses. Safe execution of this switch required implementation of an associated independent monitoring strategy, the primary objective of which was verification that tOPV was no longer available for administration post-switch. This strategy had to be both practical and rigorous such that tOPV withdrawal could be reasonably employed and confirmed in all countries and territories within a discreet timeframe. Following these principles, WHO recommended that designated monitors in each of the 155 countries and territories visit all vaccine stores as well as a 10% sample of highest-risk health facilities within two weeks of the national switch date, removing any tOPV vials found. National governments were required to provide the WHO with formal validation of execution and monitoring of the switch. In practice, all countries reported cessation of tOPV by 12 May 2016 and 95% of countries and territories submitted detailed monitoring data to WHO. According to these data, 272 out of 276 (99%) national stores, 3,741 out of 3.968 (94%) regional stores, 16,144 out of 22,372 (72%) district level stores, and 143,050 out of 595,401 (24%) of health facilities were monitored. These data, along with field reports suggest that monitoring and validation of the switch was efficient and effective, and that the strategies used during the process could be adapted to future stages of OPV withdrawal.

The Polio Eradication and Endgame Strategic Plan 2013–2018 (the “Endgame Plan”) requires cessation of use of all oral polio vaccines (OPVs) after the eradication of types 1, 2, and 3 polioviruses in order to eliminate vaccine-associated paralytic poliomyelitis (VAPP) and vaccine-derived polioviruses (VDPVs) [1]. While use of OPV has been essential to the polio eradication effort, OPV contains attenuated polioviruses that mutate during replication, and in areas with low population immunity can, in rare instances, regain the ability to cause paralytic polio. As the danger posed by wild polioviruses (WPVs) disappears with their eradication, the risks of administering OPV begin to outweigh its benefits. Because eradication of the different subtypes of WPV is occurring at different times, the removal of OPV is being conducted in phases. This phased withdrawal began with the introduction of bivalent OPV (bOPV), containing only attenuated types 1 and 3 polioviruses, in place of trivalent OPV (tOPV), containing attenuated types 1, 2, and 3 polioviruses. This transition from tOPV to bOPV (referred to as the “switch”) was conducted first because wild poliovirus type 2 (WPV2) was eradicated first, and because type 2 circulating VDPVs (cVDPV2s) have caused the vast majority of polio cases since 2006 [2, 3]. Complete cessation of OPV will occur once the remaining WPVs are eradicated. (WPV type 3 was last detected in 2012, but has not yet been officially certified as eradicated.)

While the switch was an important milestone toward polio eradication, it was not without risk. In addition to preventing creation of new VDPV2s [4], the cessation of tOPV use removed a principle source of immunity to type 2 poliovirus infections. Postswitch introduction of the attenuated type 2 polioviruses found in tOPV into a population lacking immunity to type 2 poliovirus infections could lead to prolonged transmission and replication of these vaccine polioviruses, the emergence of a cVDPV2, and potentially an associated outbreak [5]. Such an introduction could occur through ongoing cVDPV2 transmission at the time of the switch, an importation of an attenuated type 2 poliovirus from an area still using tOPV, or continued administration of tOPV in an area that had supposedly ceased tOPV use [6, 7]. These risks associated with the switch were mitigated by (1) tOPV supplemental immunization activities held prior to the switch, (2) global synchronization of switch time frames for the risk of importation, (3) extensive field monitoring aimed at validating withdrawal of tOPV from the vaccine cold chain and services points, and (4) a requirement for the destruction of tOPV to prevent the risk of continued tOPV administration after the switch [5, 8].

The need to withdraw OPV formulations containing particular serotypes in a synchronized manner accompanied by standardized global monitoring was anticipated. In 2005, Kew and colleagues [9] noted that “cessation should be coordinated among countries to be completed within a few weeks, and reliable mechanisms established in all countries to assure that all OPV stocks in the field are recalled and destroyed.” Similarly, the Endgame Plan instructed that, “following the transition from tOPV to bOPV, all remaining stocks of tOPV must be destroyed or securely stored at the national level, within 3 months. Documentation of the process of tOPV’s withdrawal from use and the collection and destruction of remaining stocks will be critical for National Certification Committees and the Regional and Global Certification Commissions” [1]. Translating these ideas into a practical switch monitoring plan fell to the Immunization Systems Management Group (IMG), a time-limited, multiagency entity cochaired by the World Health Organization (WHO) and the United Nations Children’s Fund (UNICEF), responsible for the management and coordination of the Global Polio Eradication Initiative’s (GPEI’s) efforts to introduce inactivated poliovirus vaccine (IPV), withdraw OPV, and strengthen routine immunization systems.

## RATIONALE AND APPROACH

**Figure 1.**
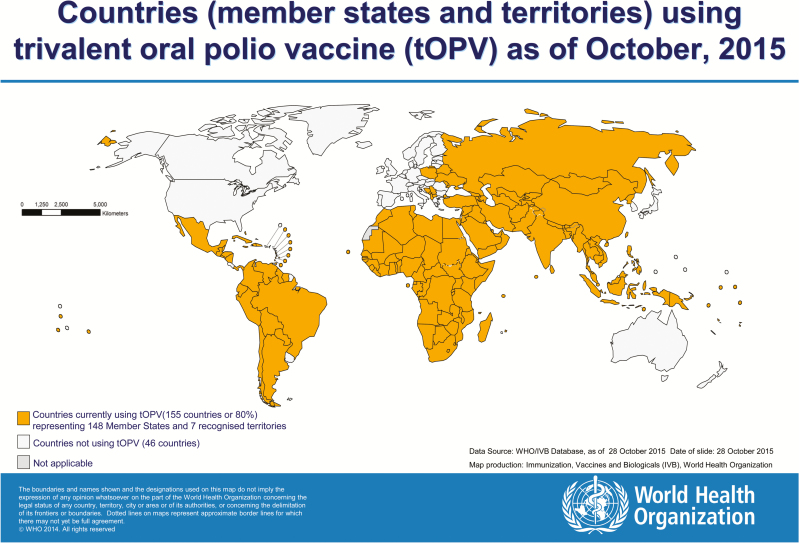
Countries (member states and territories) using trivalent oral polio vaccine (tOPV) as of October 2015.

Discussions of the monitoring component of the switch began in 2014 in conjunction with a presentation to the WHO Strategic Advisory Group of Experts (SAGE) on Immunization, and was accelerated in early 2015 with the establishment of a subgroup of the IMG, the switch working group (SWG), dedicated to switch coordination [10]. Initial planning assumed that the switch would occur globally within a 2-week time frame, and that all 155 countries and territories that were using OPV at the time (Figure 1) would be required to remove all tOPV from all cold chain stores and health facilities on a designated national switch day.

The SWG identified several goals for global switch monitoring. First, monitoring aimed to reduce the risk of emergence of new cVDPV2s postswitch by confirming that all tOPV had been removed from the vaccine cold chain and service points (a proxy indicator for discontinuation of administration of tOPV). While assessment of the availability of bOPV and IPV were ancillary goals, given the available resources, the monitoring efforts needed to be targeted (ie, would not involve data collection on a wide variety of routine immunization indicators). Second, information confirming tOPV withdrawal from the cold chain needed to be collected and validated in each country in a way that provided reassurance that postswitch use of tOPV was unlikely. Third, given that the 155 OPV-using countries and territories were collectively administering several hundred million doses of tOPV in 2015 [11], the monitoring strategy had to be comprehensive yet efficient, particularly regarding identification of priority cold chain stores and health facilities from among the hundreds of thousands of these sites that existed in these countries and territories. Finally, the monitoring plan needed to be practical, allowing its execution in a very short time frame.

To meet these goals, the SWG developed a multifaceted monitoring strategy modeled in part after GPEI’s methods for monitoring the performance of OPV supplemental immunization activities (SIAs). To verify that tOPV had been removed from the cold chain and to meet ancillary switch goals, switch monitors would visit cold chain stores and selected health facilities where they would inspect vaccine storage areas and remove any opened or unopened tOPV vials found. The potential for a monitoring visit was expected to motivate immunization staff to remove and dispose of tOPV, and the monitors’ direct inspection was expected to detect any leftover tOPV and result in its removal from the cold chain.

To document and validate the completion of switch monitoring in each country or territory, data would be collected, aggregated, and reported to an independent body, referred to as the national switch validation committee (NSVC). Countries and territories were encouraged to employ existing governmental structures responsible for certifying the absence of polio (eg, the National Certification Committee [NCC]), to establish their NSVCs. The NSVC would review the collected monitoring data and conclude whether all tOPV had been removed nationally or if additional action was needed. Once assured that all tOPV had been withdrawn, each country’s Ministry of Health (MoH) or equivalent agency would communicate NSVC findings to its WHO regional offices via its WHO country office. WHO regional offices would assess and relay this information to WHO headquarters, where the monitoring and validation progress was being tracked at the global level. Where possible, monitoring data were also shared with the UNICEF regional and country offices to support the verification of monitoring outcomes. As is the case with the monitoring of SIAs, to increase confidence in the findings of the monitors and the NSVC, both entities needed to be independent of the individuals responsible for planning and executing the switch. Additionally, organizations independent of the government (eg, WHO and UNICEF) would provide technical assistance.

**Figure 2.**
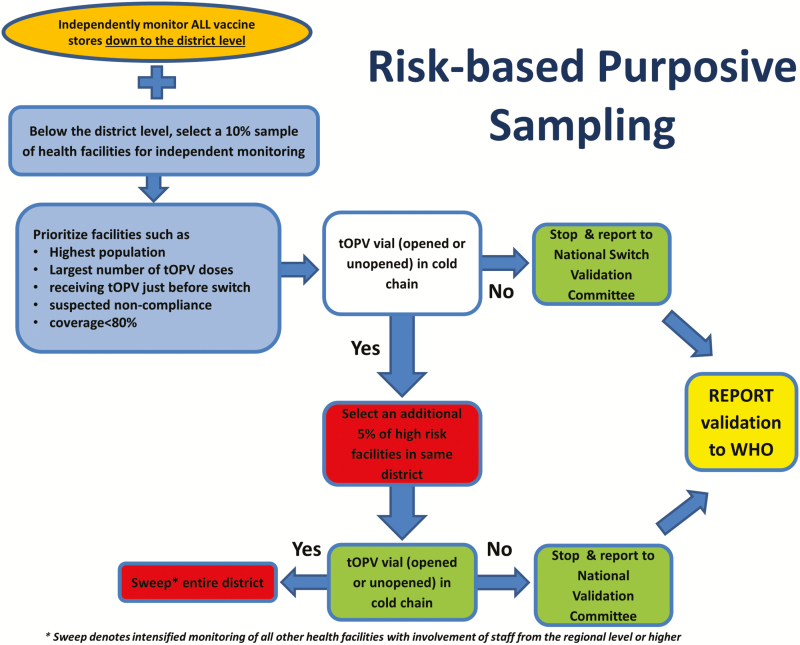
Schematic of risk-based purposive sampling.

To improve efficiency, a country’s monitors would visit all upper-level cold chain stores, where larger volumes of tOPV were likely to be found, but only a sample of health facilities at the lowest level of vaccine storage. Specifically, countries and territories were asked to monitor a sample of at least 10% of the health facilities judged by national or subnational immunization program staff to be at highest risk of continued use of tOPV postswitch. Indicators used to select facilities were based on local characteristics, and included qualitative and quantitative information (eg, facilities serving large populations, storing a large amount of vaccine, or receiving a shipment of tOPV just before the switch), as well as facilities with chronically low vaccine coverage or performance. If any opened or unopened tOPV vials were found in the cold chain in 1 or more of those facilities, an additional 5% of facilities within that district (those deemed to be at the next highest risk) would be visited. Detection of tOPV at any of these additional facilities would trigger a “sweep” (ie, monitoring of all health facilities in the district) or a comparable administrative level (Figure 2).

Given the limited number of sites that could be visited during this short time frame, and to increase confidence that tOPV had been fully withdrawn from service points, countries and territories were encouraged to integrate a continued, postswitch search for tOPV into routine supportive supervision, aimed at enhancing the quality of the WHO’s Expanded Program on Immunization (EPI). More specifically, countries and territories were advised to incorporate monitoring for tOPV into service-point visits by EPI supervisors or district surveillance officers (particularly ones not visited by independent switch monitors) as soon as possible and no later than 3 months after the national switch date. Monitoring of tOPV disposal during the switch window was encouraged, but optional. Given the practical challenges of the destruction of tOPV, particularly in countries and territories with inadequate capacity for disposal or without established policies or systems, countries and territories were given a 3-month window immediately following the switch in which to dispose of withdrawn tOPV. Table 1 provides a summary of the rationale behind and approach to switch monitoring and validation.

**Table 1.** Overview of the Rationale for and Approach to Switch Monitoring and Validation

Key considerations for monitoring and validation of synchronized tOPV withdrawal	☑ Globally implemented in 155 countries and territories between 17 April–1 May 2016 ☑ Chain and reporting and accountability is national government responsibility ☑ Completed within 2 weeks of national switch date ☑ Follows different timelines and processes than certification of poliovirus containment according to GAPIII ☑ tOPV disposal activities may take longer and would continue beyond 2 weeks depending on country situation
Risk-based monitoring strategy	☑ Reduces risk by ensuring withdrawal of tOPV from high priority sites in all countries and territories within 2 weeks of the switch date ☑ Objectives o Ensure and confirm withdrawal of tOPV from cold chain (primary) o Assess introduction of bOPV and IPV (secondary) ☑ Key components o Oversight by committee independent from staff responsible for switch planning and implementation o Use of independent monitors o Immediate corrective action ☑ Monitoring site selection o ALL national, regional, and district stores o SAMPLE of service points or health facilities with broader sweep with detection of tOPV ☑ Supplemented by ongoing routine supervisory monitoring by immunization staff
Reporting and validation	☑ Immediate corrective action to remove tOPV from cold chain ☑ Daily reporting by monitors to supervisors ☑ National supervisors prepare switch report ☑ Independent switch validation committee reviews switch report and validates switch or issues recommendation ☑ National government provides report to WHO within 2 weeks

Abbreviations: bOPV, bivalent oral polio vaccine; GAPIII, Global Action Plan III; tOPV, trivalent oral polio vaccine; WHO, World Health Organization.

## IMPLEMENTATION

**Figure 3.**
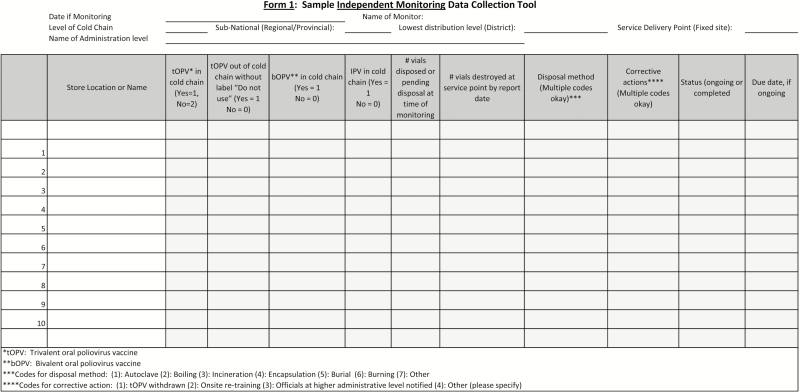
Form 1: Sample Independent Monitoring Data Collection Tool.

In October 2015, SAGE confirmed the following time frames for the switch: countries and territories were to select a date between 17 April and 01 May 2016 as their national switch day, after which the government would have 2 weeks to submit a validation report to WHO (ie, to validate the switch) [12]. In practical terms, this meant that the switch validation window ranged from 2 May to 15 May. After this endorsement, the SWG worked swiftly to finalize and disseminate national switch plan guidelines and supplementary documents (eg, tools for microplanning and developing budgets) as well as to conduct training and provide technical support for switch planning and implementation. Additionally, the SWG developed forms for monitors, NSVCs, and governments to use in collecting and reporting switch-related information (see example in Figure 3).

The WHO and UNICEF regional offices played a significant role in the development of guidelines and forms, ensuring they could be easily adapted to a country context. Presentations and practical exercises related to switch monitoring were incorporated into numerous global and regional meetings, including 5 regional switch planning workshops in the first quarter of 2016. Subsequently, WHO and UNICEF regional offices helped guide country-level switch monitoring training. The IMG also deployed 33 global observers to 21 (14%) countries to provide technical assistance, support planning, and execution of switch monitoring.

**Figure 4.**
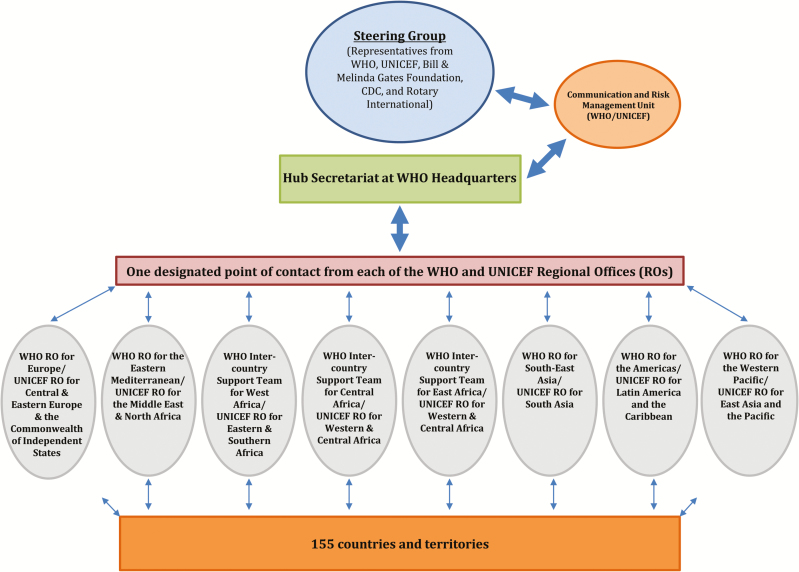
Structure of the Global Switch Information and Coordination Hub.

To track the flow of and aggregate switch-related information and receipt of country validation reports, the IMG established a time-limited Global Switch Information and Coordination Hub (the Hub) (Figure 4). The Hub aimed to compile all countries’ validation reports by 18 May (ie, after the end switch validation window, but before the 23 May meeting of the WHA). The Hub comprised a secretariat that managed the flow of information between the regional and global levels and generated daily reports; liaisons from the WHO and UNICEF regional offices who coordinated country-level updates; a communications and risk management group; and a steering group that monitored overall progress, provided guidance, and responded to any emerging issues.

## OUTCOMES

Anecdotal information from regional offices and reports from the deployed global observers suggest that the planning process for monitoring and validation activities was straightforward. Countries predominantly elected to employ their NCCs as NSVCs, but showed creativity and flexibility in recruiting monitors from a variety of local entities, (eg, UNICEF and WHO Country Offices, the MoH, nongovernmental organizations, hospitals, professional organizations, and universities). Countries and territories readily adapted IMG switch templates to the national context and developed microplans by which to sample health facilities, distribute materials, and assign and transport monitors to specific sites.

By 2 May 2016 (the start of the switch validation window), 149 (96%) out of the 155 countries and territories reported having stopped the use of tOPV, and 117 (75%) countries and territories reported having begun the monitoring process. (Monitoring was not expected in 3 countries [Israel, Malaysia, and Poland] as they had stopped use of tOPV prior to the switch and had already confirmed with the WHO regional offices that tOPV was no longer present in the countries.) By 8 May, all 155 countries and territories (with the exception of Egypt) confirmed that all tOPV use had indeed ceased by the 1 May deadline. On 12 May, when Egypt reported stopping tOPV use, switch implementation was considered complete. A majority of the countries initiated and completed monitoring within 2 weeks of their national switch date, with the last country initiating monitoring on 19 May.

**Figure 5.**
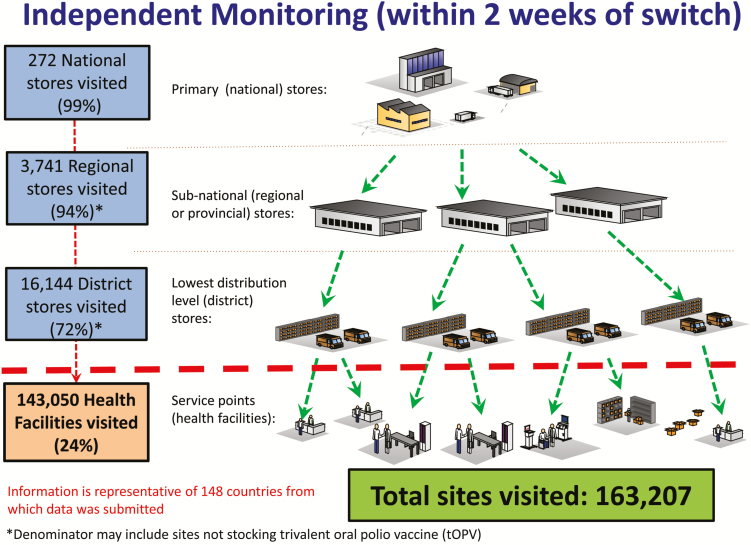
Summary of sites reported as visited during independent monitoring of the switch.

Of the 155 countries and territories, 148 (95%) submitted detailed monitoring data to WHO. According to these data, more than 270 national-level cold chain stores, 3700 regional and 16000 district cold chain stores, and 140000 health facilities were monitored worldwide (Figure 5). The global goal of monitoring 100% of cold chain stores down to the district level was largely met: 272 out of 276 (99%) national-level stores, 3741 out of 3968 (94%) regional-level stores, and 16144 out of 22372 (72%) district-level stores were reported as monitored. The goal of monitoring 10% of health facilities was far exceeded. Globally, countries reported that monitors visited 143050 out of 595401 (24%) of health facilities, and in some countries and territories (particularly small or island countries), 100% were monitored.

Out of the 155 countries and territories that provided monitoring data, tOPV was found in the national cold stores of 13 (8%), in the regional cold stores of 14 (9%), in the district cold stores of 30 (19%), and in the health facilities of 58 (37%) countries. The sites where tOPV was found, however, accounted for a small minority of the overall sites monitored, particularly at the regional-, district-, and health-facility levels. Among the countries and territories that reported finding tOPV in at least 1 monitored site at the respective level, tOPV was found on average in 73% (range, 17%–100%) of national cold stores, 11% (range, 1%–38%) of regional cold stores, 6% (range, 1%–45%) of district cold stores, and 8% (range, 1%–90%) of health facilities. All countries and territories that reported finding tOPV also reported the withdrawal and disposal of this tOPV. Monitors reported finding bOPV in 134 countries and territories (86%), and an overall average of 91% of health facilities (range, 12%–100%) were found to have bOPV. Among the 129 countries and territories that had introduced IPV by 1 May, 128 (99%) submitted monitoring data, and monitors reported finding IPV in 1 or more health facilities in 114 (88%) countries and territories.

Only 21 (14%) of the 155 countries and territories met the requirement of submission of a validation report to WHO within 2 weeks of their national switch date. By 15 May, the last day of the global validation window, however, WHO had received a national validation report from 77 (50%) countries and territories, and by the end of May, that number increased to 147 (95%). Seven additional countries provided reports by early September 2016. The report from 1 remaining country (Iraq) is still pending the destruction of the remaining tOPV that is being stored in its national cold store.

## DISCUSSION

The switch was an unprecedented global public health event, both due to its synchronization across such a large number of countries, and the short time frame in which it was completed [13]. The comprehensive monitoring and validation strategy helped assure countries that any risks provoked by the switch were being minimized, and that its execution was achievable. The monitoring strategy’s own feasibility is indicated by the high proportion of vaccine stores and health facilities that were visited. The detection of tOPV at a few monitored sites suggests that monitoring was a useful adjunct that helped reduce the likelihood of future use of residual tOPV, while the absence of tOPV from most monitored sites indicates that monitoring of the switch did not overly distract from the actual switch process.

Balancing the goal of risk reduction with the goal of effective monitoring, however, was challenging. Confirming the complete elimination of the risk of tOPV use postswitch would have required field monitors to visit every cold chain store and health facility within each country. This approach, however, would have been inefficient and impractical to conduct within the 2-week window, especially for large countries. Conversely, monitoring only vaccine stores and excluding health facilities would have been easier and more practical. As vaccine stores hold the greatest volumes of tOPV, monitoring them would prevent further systemic distribution of vaccines to lower levels. This approach, however, would not have provided reassurance that tOPV had been removed from health facilities where, even if quantities were small, the risk of actually administering tOPV after the switch and potentially causing a cVPDV2 was the greatest.

Ultimately, there were tradeoffs related to the adequacy of reporting monitoring results. GPEI’s strategic plan emphasizes that the results of switch monitoring be geared toward the needs of the polio NCCs and Regional and Global Certification Commissions (RCCs and GCCs), and that monitoring be as stringent as possible in order to provide the maximum reassurance of global withdrawal of tOPV. In other words, either all cold stores and health facilities that might have OPV should be monitored, or all cold stores and a random sample of health facilities that would be representative of all health facilities should be monitored. Similar approaches were used in WHO’s Western Pacific Region from 1999 to 2008 to verify containment or destruction of all WPVs, and in WHO’s American Region from 2001 to 2010 to verify containment or destruction of all WPVs, respectively [14, 15]. Given the lengthy process and high cost of conducting regional inventories and lower risk of seeding outbreaks from attenuated tOPV as compared to WPV, however, such a stringent approach was deemed impractical. Instead, each country’s MoH was asked to take responsibility for validating its own switch.

Delinking switch monitoring and validation from the polio certification process facilitated the implementation of a purposive sampling approach to selecting health facilities for monitoring that was more compatible with the goal of completing switch monitoring work within 2 weeks of the national switch date (Table 1). This approach, which was not intended to provide information generalizable to health facilities not included in the sample, is similar to that used for selecting surveys areas for validation of elimination of maternal–neonatal tetanus (MNT) from a given country. As with switch validation, validation of MNT elimination involves monitors collecting data regarding a country’s fulfillment of prespecified criteria for validation, but does not require an international body equivalent to the polio RCCs or GCC to review that data and confirm that a country has eliminated MNT [16].

Nearly all countries and territories complied with global monitoring recommendations and submitted switch validation reports to WHO. Half of the countries and territories submitted their validation reports by the end of the 2-week validation window, whereas all but 2 reports were received within a month of the start of the validation period. Several countries and regions still interpreted 15 May (the last day of the switch window) to be a general deadline for validation, regardless of country switch date. Future validation efforts (eg, for bOPV withdrawal) should expect some delays in reporting, and discussions regarding time lines should weigh the pros and cons of establishing a single global deadline for submission of validation reports versus a staggered validation window following the national date of implementation.

Despite the generally successful execution of the switch, the risk of VDPV2s arising from use of tOPV after the switch has not been eliminated entirely. Because monitors reported finding some residual tOPV in numerous countries, and not all countries submitted detailed validation reports, the number of tOPV vials found may underestimate the actual number of vials that remain globally. Although any undiscovered stocks of stored tOPV are likely quite small, they could still exist. Also, the quality and completeness of national monitoring data varied considerably, and GPEI’s ability to verify the information contained in the monitoring reports was limited. Because the health facilities monitored in many countries were a nonrepresentative sample, the findings from the sampled health facilities cannot be generalized to health facilities that were not monitored. A practical purposive sampling strategy was chosen to substantially reduce the risk within a few weeks of the switch, but it did not eliminate all risk. Thus, countries were encouraged to continue ongoing inspection of sites through routine, supportive supervisory visits in the months following the switch.

As evidenced by WHO’s America Region’s 2-phase monitoring process, ongoing supportive supervision can help further reduce the minimal risk associated with residual tOPV [17]. In addition to complying with the IMG’s switch monitoring requirements, the WHO American Regional Office asked its countries and territories to conduct monitoring in 100% of facilities by the end of July. During this second phase, additional, although limited, quantities of tOPV were indeed found in some countries. While the vast majority of these additional vials had already been withdrawn from the cold chain, the results underscore the value of continued routine monitoring and supportive supervision. For future OPV withdrawals (especially the final phase), experts should explore whether requiring 100% of health facilities to be monitored as part of supportive supervision within a specific time frame, in addition to a minimum standard for independent monitoring, is potentially a reasonable and effective way to confirm the total withdrawal of OPV.

Certified poliovirus-essential facilities that continue to handle and store OPV2/Sabin 2 materials, and the monovalent type 2 oral polio vaccine (mOPV2) stockpile that has been amassed at the global level in case of future VDPV2 outbreaks are 2 additional sources of VDPV2 risk [4]. Fortunately, the Global Polio Laboratory Network (GPLN) is equipped to detect both VDPVs and the Sabin-strain polioviruses found in tOPV, to determine virus type and to estimate via genetic sequencing when the tOPV of origin was used [18–20]. If the switch was completely successful in stopping tOPV use, GPLN should stop detecting Sabin strain type 2 polioviruses within a few months of the switch [21–25]. Detection of Sabin strain type 2 poliovirus more than 4 months after the switch should prompt an investigation regarding possible continued tOPV use, potentially including searches for tOPV in the surrounding area [26].

In September 2016, Sabin-like type 2 polioviruses were detected in Hyderabad and Ahmedabad, India, through environmental surveillance. Subsequent investigations discovered multiple vials of opened and unopened tOPV in private clinics and vaccine retailers in the 2 cities, but none in government clinics or vaccine stores. The detection of the Sabin-like type 2 polioviruses and subsequent finding of tOPV highlights the importance of continued surveillance for Sabin-like type 2 polioviruses (particularly environmental surveillance), and indicate that future investigations triggered by the detection of Sabin-like type 2 polioviruses should carefully assess the private sector.

While the global withdrawal of tOPV provides a good model for future vaccine withdrawals (eg, the global withdrawal of bOPV or the withdrawal of mOPV2 following a campaign), these withdrawals will occur under different circumstances and conditions. The switch monitoring plan assumed that a robust response would be possible in the event of an outbreak. If a future OPV withdrawal occurs in the context of diminished polio outbreak–response capacity, however, it may be worthwhile to monitor a larger proportion of health facilities, or a more representative sample. More rigorous monitoring of the private sector may also be in order, based on the finding of tOPV in Indian private clinics and vaccine retailers in September 2016. The level of stringency of monitoring associated with the withdrawal of all OPV will depend on the level of risk deemed acceptable by experts, GPEI partners, and countries at that time. The global community may have a lower acceptance of risk after polio eradication and the ultimate withdrawal of OPV, thus warranting a more stringent approach to monitoring than was recommended for OPV2 withdrawal. It is worth noting, however, that in the past when tOPV was widely used, Sabin strain types 1 and 3 polioviruses were somewhat less likely to evolve into VDPV1s and VDPV3s than Sabin strain type 2 polioviruses are to evolve into VDPV2s [9]. If bOPV is widely used in the period leading up to bOPV withdrawal, and if transmission of cVDPVs 1 and 3 has been negligible for a number of years, a less stringent monitoring approach and a longer window of OPV cessation may be acceptable.

In conclusion, both monitoring data and anecdotal field reports suggest that the switch was implemented successfully, particularly with regard to the comprehensive withdrawal of tOPV. The switch monitoring strategy was feasible and identified small amounts of tOPV in numerous countries that remained in the cold chain after the switch. This strategy provides an adaptable model for monitoring future withdrawals of OPV. Ongoing supportive supervision, assiduous laboratory surveillance for VDPV2, extensive training on and strict adherence to VDPV2 outbreak protocols, and prompt destruction of any tOPV identified postswitch will be essential to keeping VDPV2 risk at a minimum level now and during future stages of OPV withdrawal.

## Notes

***Disclaimer.*** The findings, interpretations, and conclusions expressed in this paper are those of the authors and do not necessarily reflect the policies or views of their respective organizations.

***Financial support***. This work was supported by the authors’ affiliated institutions, including United Nations Children’s Fund, Centers for Disease Control and Prevention, and World Health Organization. Some of the authors are supported by a grant from the Bill & Melinda Gates Foundation (M. F., J. G., S. W., and M. M. P.).

***Supplement sponsorship.*** This work is part of a supplement coordinated by the Task Force for Global Health with funding provided by The Bill and Melinda Gates Foundation and the Centers for Disease Control and Prevention.

***Potential conflicts of interest.*** All authors: No reported conflicts. All authors have submitted the ICMJE Form for Disclosure of Potential Conflicts of Interest. Conflicts that the editors consider relevant to the content of the manuscript have been disclosed.
